# Brain mechanisms linking language processing and open motor skill training

**DOI:** 10.3389/fnhum.2022.911894

**Published:** 2022-08-04

**Authors:** Yixuan Wang, Qingchun Ji, Chenglin Zhou, Yingying Wang

**Affiliations:** ^1^School of Psychology, Shanghai University of Sport, Shanghai, China; ^2^Department of Physical Education, Shanghai University of Engineering Science, Shanghai, China

**Keywords:** language processing, open motor skill training, fMRI, visual regions, table tennis players

## Abstract

Given the discovery of a distributed language and motor functional network, surprisingly few studies have explored whether language processing is related to motor skill training. To address this issue, the present study used functional magnetic resonance imaging to compare whole-brain activation between nonexperts and experts in table tennis, an open skill sport in which players make rapid decisions in response to an ever-changing environment. Whole-brain activation was assessed in 30 expert table tennis players with more than 7 years’ experience and 35 age-matched nonexpert college students while they performed both a size and a semantic judgment task of words presented on a monitor. Compared with nonexperts, expert table tennis players showed greater activation in the left middle occipital gyrus and right precuneus while judging the size of the words versus during baseline fixation. They also showed greater activation in the left lingual gyrus during the semantic judgment task versus during baseline fixation. Our findings indicate that the visual regions engaged in language processing are associated with open motor skill training.

## Introduction

There is a growing body of evidence in the literature emphasizing the mutual coordination of language and motor systems in the brain, although these systems have different cortical bases in circumscribed areas (i.e., left hemisphere “language regions” vs. motor cortex) underlying linguistic functions and body movement, respectively (Pulvermüller, 2005; Vukovic et al., 2017; Dreyer and Pulvermüller, 2018; Tian et al., 2020; Bhroin et al., 2021). Previous studies have found that motor activity is involved in language comprehension and that semantic representation is involved in action processing (Vukovic et al., 2017; Vukovic and Shtyrov, 2019; Wang et al., 2019). The cortical systems for language and motor processing have developed into a distributed functional network that allows for the processing of both language and motor information (Pulvermüller, 2005; Papitto et al., 2020). For example, the embodied language theory proposes that language processing is carried by neuronal circuits distributed across multimodal areas that include the sensorimotor cortex (Willems and Casasanto, 2011; Dreyer and Pulvermüller, 2018). However, given the plasticity of the brain, the function and structure of the motor system may change in expert athletes after years of training. How such altered motor systems play a role in language processing still requires investigation.

The classic “embodied empiricism” asserts that knowledge can be acquired through visual, auditory, motor, and other sensory-motor experiences. Knowledge derived from sensory-motor experience is stored in sensory-motor association cortices (Barsalou et al., 2003; Martin, 2016). Expert players of open skill sports, that is, sports in which players must react to unpredictable and fast-changing environments, such as table tennis, provide a helpful model to assess this assertion. Players engaged in motor activity of open skill sports within their expertise must continuously process the opponents’ different ball striking actions and return the ball. The sensory-motor experience achieved by players may be stored in their sensory and their motor cortices. Neuroimaging studies have shown structural and functional plasticity in both visual and motor systems related to long-term professional motor skill training (Wright et al., 2011; Balser et al., 2014; Zhang et al., 2021). Many behavioral studies have demonstrated that compared with less-experienced or nonexpert players, expert players with rich motor experience better process other players’ actions (Ward et al., 2002; Aglioti et al., 2008; Williams et al., 2009; Causer et al., 2017; Li and Feng, 2020) when the motor system shows increased activation (Wright et al., 2011; Balser et al., 2014; Wang et al., 2019). Although superior motor activity and consequent superior action processing in expert players has been described, little is known about their ability to process language through the coordination of language and motor systems.

In sports, players are given verbal instructions for actions from their coaches during motor skill training. A link between a specific action and the corresponding instruction is established and is gradually stabilized with the acquisition of the motor skill. Therefore, motor learning involves a strong functional association between motor performance and somatosensory and language feedback. When players execute or observe actions within their expertise, the related linguistic information is activated (Gentilucci et al., 2004). Beilock et al. (2008) found that the more hockey training experience individuals had, the more effective they were in comprehending hockey-related sentences. Language is also necessary to generate, select, and implement higher-level action plans (Lindemann et al., 2006). The two-pathway framework by Kilner (2011) proposed that the highest level people describe actions is the intention level, which encodes the most abstract semantic representations (Kilner, 2011). In the context of interceptive sports, expert players usually understand the meaning of their opponents’ actions to predict future movements and to implement a selected action plan. Our previous studies have found that compared with nonexperts, action processing in table tennis players engages both semantic and sensory-motor regions and action words impair their action processing (Wang et al., 2019, 2022). Whether expert table tennis players with open skill training experience and nonexperts without any sport training experience show different behavioral and brain responses during language processing is unclear.

We here compared behavioral and brain responses in expert table tennis players to those in nonexperts when performing a word judgment task to examine the association between language processing and open motor skill training. In this task, participants completed both semantic and perceptual size judgments of Chinese nouns in order to exclude verb-induced motor cortical activation and to purely explore the activation of language regions related to sensory-motor experience. We hypothesized that expert players would outperform nonexperts on the word judgment tasks. We further expected that the semantic processing of words would increase activation of the language regions in expert players to a greater extent than that in nonexperts given the results from our previous study assessing the classic semantic brain network, which includes the posterior inferior parietal lobe (angular gyrus), middle temporal gyrus, fusiform and parahippocampal gyri, dorsomedial prefrontal cortex, inferior frontal gyrus, ventromedial prefrontal cortex, and posterior cingulate gyrus (Binder et al., 2009; Wang et al., 2019).

## Materials and methods

### Participants

Thirty expert table tennis players and a control group of 35 college students who had no professional training in table tennis and no other sports experience were recruited from the Shanghai University of Sport in Shanghai, China (Table 1). The expert players had more than 7 years of table tennis training. Experts and nonexperts did not significantly differ in age (*t*_(63)_ = 1.60, *p* = 0.115, 95% confidence interval, -0.164 to 1.478) or sex ratio (χ^2^ = 0.15, *p* = 0.702). All participants had normal or corrected-to-normal vision and had no history of psychiatric, medical, or neurological illness. All participants provided written informed consent prior to the study. The experimental protocol was approved by the Ethics Committee of Shanghai University of Sport.

**TABLE 1:** Participant demographic and training characteristics.

Characteristic	Experts	Non-experts
Number	30	35
Sex, (No. males/females)	14/16	18/17
Age, years, mean ± SD	20.00 ± 1.68	20.66 ± 1.63
Years of training, mean ± SD	12.13 ± 2.56	none
Training frequency (No. of sessions/week), mean ± SD	4.93 ± 3.34	none
Training Time (h/session), mean ± SD	2.23 ± 0.61	none

### Stimulus and task

**FIGURE 1:**
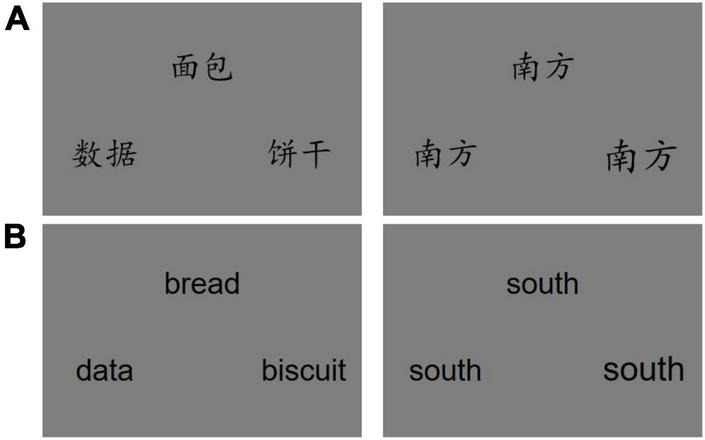
Example stimuli for the semantic and size judgment tasks. Left panels show the stimulus triplets in the semantic judgment task in which participants were instructed to respond by matching the meaning of one of the two words presented at the bottom of the monitor to that of the sample word presented at the top. The right panels are exemplars of the size judgment task. Panels in **(B)** are the English translation of the Chinese words presented in **(A)**.

Participants performed a block design, language functional magnetic resonance imaging (fMRI) experiment programmed with E-Prime software (Psychology Software Tools, Pittsburgh, PA) involving either semantic or perceptual size judgment of Chinese words. All the words were two-character nouns presented on a monitor. They were matched for valence, arousal, imageability, and word frequency across the two tasks (Table 2). The matching was determined by our pilot testing of an independent sample of 10 native-Chinese speakers, who used a semantic rating procedure with a five-point scale. In the semantic judgment task, three different words were presented, and participants were instructed to choose the item closest in meaning to the sample stimulus (left panels in Figure 1). In the size judgment task that served as the control condition, three identical words were presented, and one of them was 14% larger than the sample item (right panels in Figure 1). Participants were instructed to choose the item that was closest in size to the sample.

**TABLE 2:** Psycholinguistic properties of the words in semantic and perceptual size judgment tasks (mean ± standard deviation).

Psycholi nguistic feature	Semantic judgment task	Size judgment task	*z score*	*p value*
Valence	3.40 ± 0.39	3.35 ± 0.44	−1.020	0.308
Arousal	3.30 ± 0.36	3.21 ± 0.44	−1.361	0.174
Imageability	4.11 ± 0.70	4.08 ± 0.78	−0.301	0.763
Word frequency	4.00 ± 0.38	3.91 ± 0.34	−1.633	0.102

Differences in psycholinguistic features of words used between the semantic and size judgment tasks were assessed using the Wilcoxon signed rank test.

**FIGURE 2:**
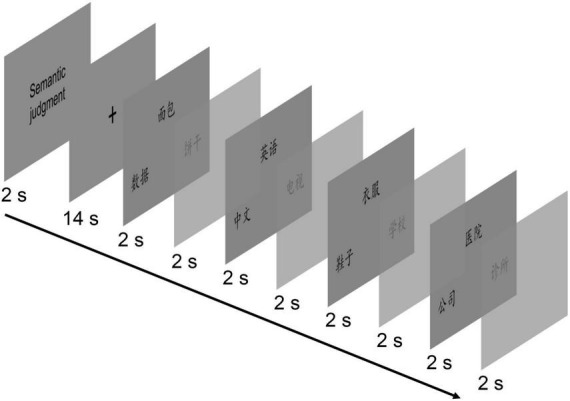
Sequence and timing of events within a single block of the semantic judgment task. The same sequence and timing of events were used in the size judgment task.

Both the semantic and size judgment tasks comprised 32 stimulus triplets that were randomly assigned to 8 blocks (for a total of 16 blocks). Thus, each block included four triplets (Figure 2). The order of the task blocks was counterbalanced. Each block began with the corresponding instruction lasting for 2.0 s, followed by a fixation cross for 14.0 s (together, equivalent to one block). Four continuous trials were then presented in which the word triplets were displayed for 2.0 s and a response blank was displayed for 2.0 s. Responses were given by pressing the corresponding button on a two-button pad. Participants practiced before they underwent scanning to become familiar with the tasks.

### Magnetic resonance imaging data acquisition

Magnetic resonance imaging (MRI) acquisition was performed using a 3T scanner (GE Discovery MR750 3.0 T scanner, GE Medical Systems, Waukesha, WI). Functional images were acquired using a gradient echo-planar imaging sequence (repetition time, 2000 ms; echo time, 30 ms; 43 slices; voxel size, 3.44 × 3.44 × 3.2 mm^3^; interslice gap, 0 mm; fractional anisotropy, 90°; field of view, 220 × 220 mm^2^). A T1-weighted anatomical MRI was also acquired (repetition time, 8.156 ms; echo time, 3.18 ms; 176 slices; voxel size, 1 × 1 × 1 mm^3^; fractional anisotropy, 8°; field of view, 256 × 256 mm^2^).

### Data analysis

#### Behavioral data analysis

We calculated the percentage of correct responses (accuracy) and the response time (RT) for both the semantic and perceptual size judgment tasks. We then conducted an arcsine transformation of the accuracy data (Hogg and Craig, 1995). Both arcsine-transformed accuracy and RT were assessed using a two-way mixed-model analysis of variance (ANOVA), with group (expert vs. novice) as the between-subject factor and task (semantic judgment and size judgment) as the within-subject factor.

Statistical analysis was performed using SPSS, version 20.0 (IBM SPSS, Inc.; Chicago, IL, United States). The *post hoc* tests of significant main effects were corrected using Bonferroni corrections. A simple effects test, which also used Bonferroni corrections, was conducted when the interaction was significant. All tests were two-sided, and *p* < 0.05 was considered statistically significant. Partial eta-squared ($\eta_{p}^{2}$) values are reported to demonstrate the effect size in the ANOVA.

#### Functional magnetic resonance imaging data analysis

Functional imaging data were preprocessed and analyzed using Data Processing Assistant for Resting-State fMRI software (DPARSF^1^) (Yan and Zang, 2010), including slice timing, head motion correction, normalization to individual participant’s T1-segmented anatomical scans with a resolution of 3 mm × 3 mm × 3 mm, and smoothing with an isotropic Gaussian kernel of 6 mm full width at half maximum.

For each participant, a general linear model analysis was performed to statistically assess the preprocessed images with a canonical hemodynamic response function at the onset of each word triplet by using Statistical Parametric Mapping software (SPM8^2^). Head movement estimates were included in the general linear model as regressors. The data and model were high-pass filtered to a cutoff of 128 s. After model estimation, the task-related T-contrasts were defined in the first-level analysis: (i) size task > fixation cross, (ii) semantic task > fixation cross, and (iii) semantic task > size task. The resulting contrast images, which reflected the intensity of brain activation for each participant, were subjected to a second-level (group-level) analysis using one-sample *t*-tests for each group and independent-sample *t*-tests (expert players vs. nonexperts) at the whole brain level. Activation maps were corrected using a Gaussian random field, with *p* < 0.05 at the voxel level and *p* < 0.05 at the cluster level indicating statistically significant differences (Liu et al., 2019; Zheng et al., 2021).

To more directly assess the difference between expert athletes and nonexperts in activity across the language regions, we used an a prior anatomical hypothesis and defined regions of interest (ROIs) based on a meta-analysis of semantic processing to comprise the following seven brain regions with an established role in semantic analysis: the posterior inferior parietal lobe (angular gyrus), middle temporal gyrus, fusiform and parahippocampal gyri, dorsomedial prefrontal cortex, inferior frontal gyrus, ventromedial prefrontal cortex, and posterior cingulate gyrus (Binder et al., 2009; Wang et al., 2019). Using the MarsBaR region of interest toolbox,^3^ the mean percentage signal changes in these seven language regions were obtained. For each region, a group × task ANOVA model was used to indicate the extent to which a difference in activity in these areas when completing semantic and perceptual size judgments of nouns varied between groups.

## Results

### Behavioral results

For arcsine-transformed accuracy, the ANOVA analysis showed a significant main effect of task (*F*_(1,63)_ = 18.435, *p* < 0.001, $\eta_{p}^{2}$ = 0.226); accuracy was lower in the semantic judgment task [mean (M) = 0.96, standard deviation (SD) = 0.05] than in the size judgment task (*M* = 0.98, SD = 0.03). The main effect of group and the interaction of group × task were not statistically significant (all *p* > 0.05).

For RT, the ANOVA analysis also showed a significant main effect of task (*F*_(1,63)_ = 419.451, *p* < 0.001, $\eta_{p}^{2}$ = 0.869); RT was longer in the semantic judgment task (*M* = 1424.30, SD = 307.32) than in the size judgment task (*M* = 1086.87, SD = 331.24). The main effect of group and the interaction of group × task were not statistically significant (all *p* > 0.05).

### Functional magnetic resonance imaging results

#### Size task > fixation cross

**FIGURE 3:**
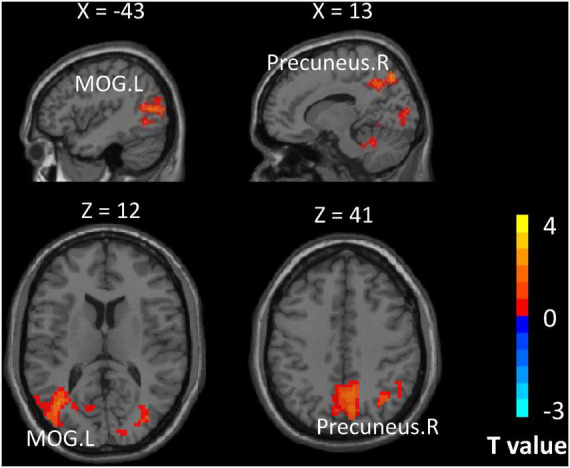
Brain regions showing differences in activation for expert table tennis players (size task minus fixation cross) compared with nonexperts (size task minus fixation cross). MOG.L indicates left middle occipital gyrus; Precuneus.R, right precuneus. Color bar indicates *t* values.

The results of the whole-brain analysis are given in Table 3. The analysis of group differences indicated stronger activation in the left middle occipital gyrus and right precuneus for expert table tennis players than for nonexperts (Figure 3).

**TABLE 3:** Brain regions showing significant between-group differences in the size judgment task.

Region	Cluster size (voxels)	T value	MNI coordinates		
			*X*	*Y*	*Z*
Left middle Occipital gyrus	162	3.03	−43	−70	11
Right precuneus	180	3.32	13	-72	49

MNI represents Montreal Neurological Institute.

#### Semantic task > fixation cross

**FIGURE 4:**
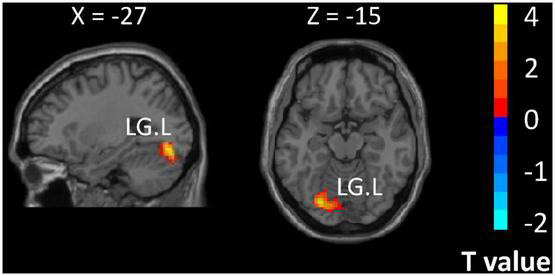
Regions showing differences in activation for expert table tennis players (semantic task minus fixation cross) compared with nonexperts (semantic task minus fixation cross). LG.L indicates left lingual gyrus. Color bar indicates *t* values.

The results of the whole-brain analysis along with the analysis of group differences indicated stronger activation in the left lingual gyrus among expert table tennis players than among nonexperts (cluster size, 123 voxels; T value, 4.03; MNI coordinates: X, -27; Y, -78; and Z, -14) (Figure 4).

#### Semantic task > size task

No significant between-group differences were found at the whole brain level in the semantic task compared with the size task.

#### Regions of interest analysis

To further explore whether there was a significant group by task interaction for language regions, we conducted an ROI analysis. The intensity of the activation for all participants in each task was extracted from ROIs and was entered into a 2 (group) × 2 (task) repeated-measures ANOVA. Only significant main effects of task were found in the middle temporal gyrus (*F*_(1,63)_ = 12.175, *p* = 0.001, $\eta_{p}^{2}$ = 0.162), inferior frontal gyrus (*F*_(1,63)_ = 62.750, *p* < 0.001, $\eta_{p}^{2}$ = 0.499), and posterior cingulate gyrus (*F*_(1,63)_ = 19.755, *p* < 0.001, $\eta_{p}^{2}$ = 0.239). In those ROIs, the activation during the semantic judgment task was higher than that during the size judgment task, but no main effect of group or an interaction between group and condition was found. No significant effects were found for the other ROIs.

## Discussion

The aim of this investigation was to identify the brain activation of language processing associated with open motor skill training. Inspired by the discovery of the distributed language and motor functional network and the characteristics of open motor skills, we compared neural responses for word processing in expert table tennis players with nonexperts. Inconsistent with our hypothesis, experts showed higher activation than nonexperts in three brain regions that were related to visual processing, including the left lingual gyrus in the semantic task and the left middle occipital gyrus and the right precuneus in the size task, compared with the fixation baseline. No classical language regions showed significant differences (Binder et al., 2009; Price, 2012).

The behavioral performance results showed a clear semantic effect in terms of both accuracy and RT. Compared with the size judgment, the semantic judgment of words took longer and was more difficult for both table tennis players and nonexperts. This finding suggested effective task manipulation. Although this finding is novel in that we found no previous research in the sports literature with similar task settings, it is in full accordance with the language literature for the well-established semantic effect related to cognitive load (Chee et al., 2000, 2001). However, there was a lack of behavioral significance between the groups. Because the mean accuracy of each group is closed to 100%. Both the size and semantic judgment tasks were too easy, and a ceiling effect was observed. Further studies increasing the task difficulty level are needed to explore the difference between expert players and nonexperts in language processing requiring more attentional resources.

In our imaging results, stronger activation was observed in two regions associated with visual processing for expert table tennis players compared with nonexperts. Specifically, activation in the left middle occipital gyrus and the right precuneus was stronger in experts than in nonexperts for the size judgment vs. fixation comparison. The occipital lobe contains most of the anatomical region known as the visual cortex, which contributes to visual information processing. The middle occipital gyrus appears to be associated with category-selective attention (Tu et al., 2013), in line with the task instruction requiring participants to classify the words at the perceptual level.

The precuneus is the central hub of the default mode network (Utevsky et al., 2014), which is typically deactivated during tasks compared with rest. Tasks that demand much attention are associated with decreased activity in the default mode network (Mazoyer et al., 2001). This suggests that compared with nonexperts, experts may use fewer attentional resources in the size judgment task. Moreover, the precuneus is connected to parietal areas and is associated with the processing of visuospatial information (Selemon and Goldman-Rakic, 1988). In the present study, these two regions were recruited as a result of increased plasticity for visual attention in experts consequent to their acquisition of motor skill training. Previous studies have found that players of table tennis, an open motor skill sport, show superior cognition function in visual reaction speed (Bhabhor et al., 2013), action perception accuracy (Klein-Soetebier et al., 2020; Schaefer and Scornaienchi, 2020), and attention control (Elferink-Gemser et al., 2018; Faber et al., 2019). These findings indicate that open motor skill training may increase functional plasticity of regions related to visual attention. In addition, increased activation of the precuneus has also been linked with language-related tasks at word-level comprehension (Kouider et al., 2010). Although the precuneus is not included in the classical semantic system, it is activated during language processing (Price, 2010). Taken together with the relationship between the activity in the precuneus and attention demand, the observed activation of the precuneus among experts during the size judgment of words also indicated enhanced attention efficiency in language processing.

A key finding in the present study is the activation of the left lingual gyrus in table tennis experts during semantic judgment vs. during baseline fixation. It was surprising that significant activation was detected in the visual cortex at the lingual gyrus only during semantic processing, not during the size judgment task. It is possible that the visual representation of Chinese characters was accessed automatically. Our results suggested that expert table tennis training may facilitate visual attention to information not only within sports expertise but also for language unrelated to sports. Sports expertise appears to produce differences in semantic-related visual processing. We speculate that this pattern of activation in visual regions influenced by expertise serves as a network to make language processing more efficient in expert players, and reflects quite a different system from the general semantic network. It is, however, important to note that the words used in our study were unrelated to sports, and further studies are needed to explore language processing within players’ expertise to further explore the association between language processing and open motor skill training.

Although previous research mainly focused on expert performance in a sport-specific or ecologically valid context, several studies reported that expert players are superior to nonexperts in basic cognitive functions (Vestberg et al., 2012; Huijgen et al., 2015; Lundgren et al., 2016; Verburgh et al., 2016). A rich sports experience could result in more efficient brain networks both in general and specific to sports (Scharfen and Memmert, 2019). But there are studies opposing the notion that expert players have superior cognitive functions (Furley and Memmert, 2010; Heppe et al., 2016). Based on our previous work exploring semantic activity related to motor experience, we expected to find in the present study a significant difference between expert players and nonexperts in the activation of language regions during language processing. However, our results showed that most activation was in the visual cortex, which is likely attributable to the sensory-motor experiences of expert table tennis players. No significant differences were observed between groups in the behavioral performance of both word judgment tasks. This finding is inconsistent with the notion of the general superiority of expert players in terms of language processing, at least for easy semantic and size judgment of words. We suggest that future studies increase task difficulty level so that language processing is not automatic and requires more attentional resources.

The classical semantic regions that constituted the ROIs in present study were derived from a meta-analysis (Binder et al., 2009) and are associated with the processing of spoken or written words. However, we found no group differences or interactions with word judgment tasks in our ROI analysis. This finding suggested that classical semantic regions linking language processing were not related to open motor skill training and added validity to the results of the whole-brain analysis.

## Conclusion

In conclusion, our findings shed light on the involvement in language processing of brain regions typically associated with visual processing, including the left middle occipital gyrus, the right precuneus, and the left lingual gyrus, among experts in sports requiring open motor skills. This functional plasticity of their visual processing regions may be due to the high demands on visual attention during long-term performance of open motor skills.

## Data availability statement

The raw data supporting the conclusions of this article will be made available by the authors, without undue reservation.

## Ethics statement

The studies involving human participants were reviewed and approved by the Ethics Committee of Shanghai University of Sport. The patients/participants provided their written informed consent to participate in this study.

## Author contributions

YXW and YYW: drafting of manuscript, analysis of data, interpreting, and validation of the results. YYW and QJ: study conception and design and acquisition of data. CZ: study conception and design, visualization, and supervision. YYW: design of paradigms and data processing and project administration. All authors contributed to the article and approved the submitted version.

## Footnotes

1
http://rfmri.org/DPARSF

2
http://www.fil.ion.ucl.ac.uk/spm

3
http://forge.net
